# Supplementary material for: A Small Molecule Targeting Human MEK1/2 Enhances ERK and p38 Phosphorylation under Oxidative Stress or with Phenothiazines
Source: Life (Basel). 2021 Mar 31;11(4):297. doi: 10.3390/life11040297 (PMC8066054; doi:10.3390/life11040297)
Supplement: Supplementary file 1 [file life-11-00297-s001.zip › life-1140876 supp.pdf]

Supplementary Material

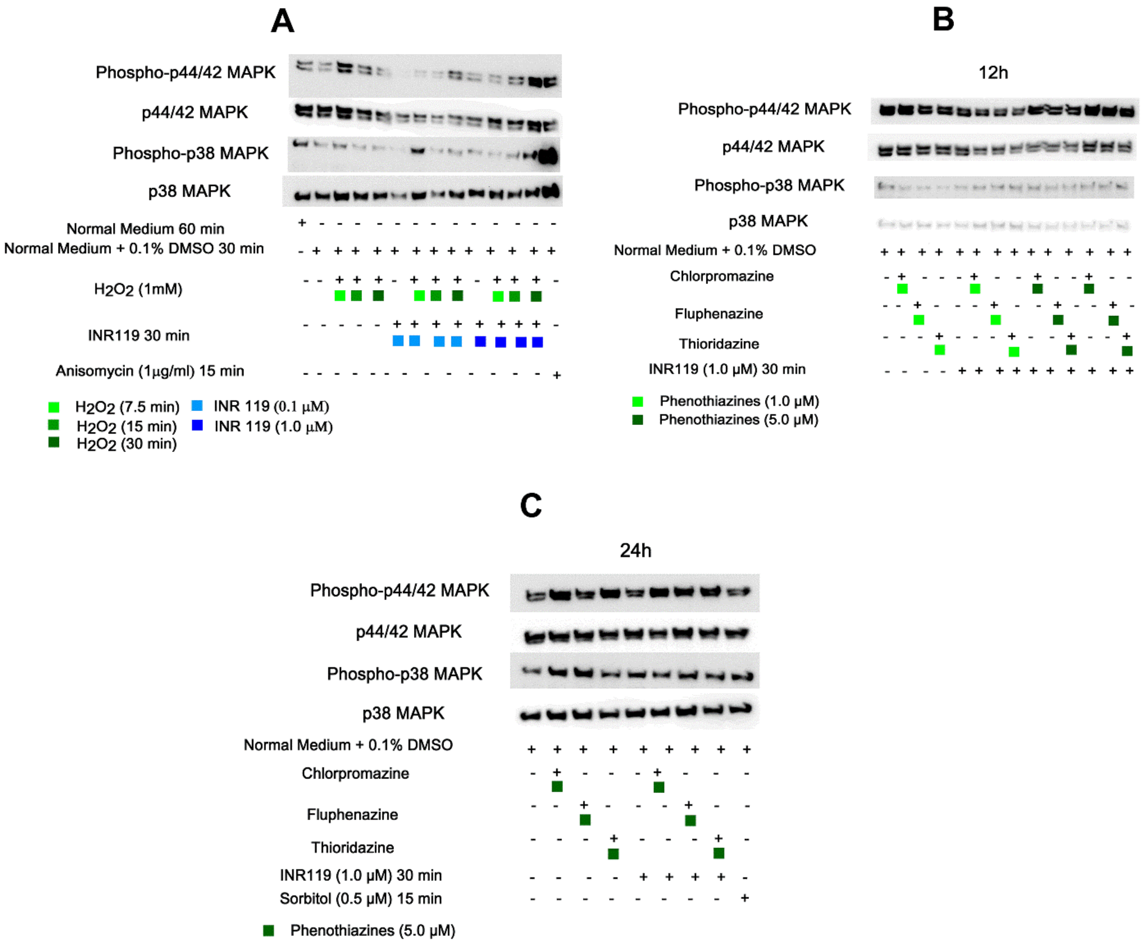

Figure S1. Western blots.

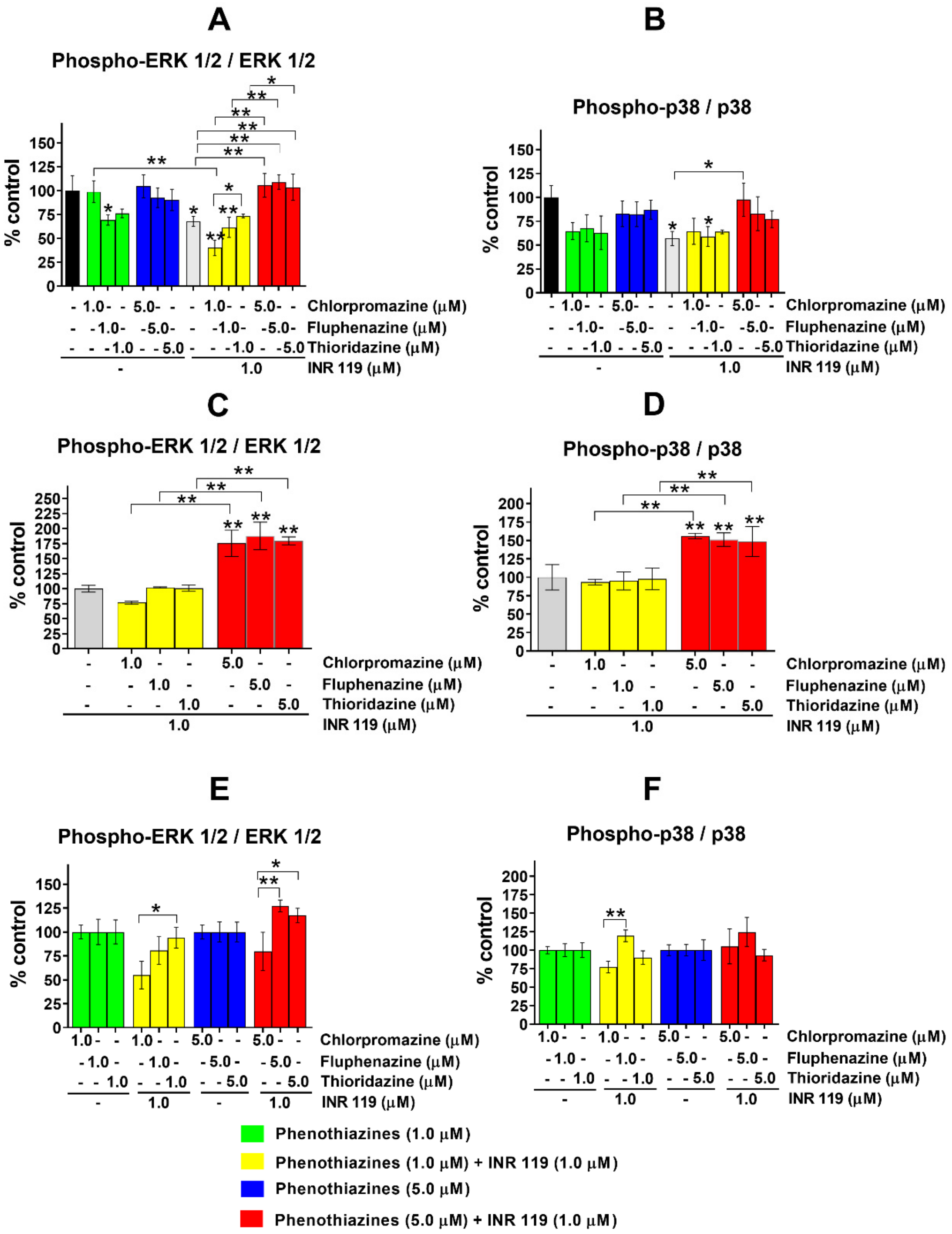

Figure S2. Phosphorylation of ERK1/2 and p38 under phenothiazine-induced oxidative stress after 12h. **(A)** Impact of phenothiazines with and without INR119 on phosphorylation of ERK1/2. **(B)** Impact of phenothiazines with and without INR119 on phosphorylation of p38. **(C)** Effect of phenothiazines on phosphorylation of ERK1/2 relative to control (medium + DMSO 0.1% + INR 119 1.0 µM). **(D)** Effect of phenothiazines (1.0 or 5.0 µM) on the phosphorylation of p38 relative to control (medium + DMSO 0.1% + INR 119 1.0 µM). **(E)** Effect of INR119 (1.0 µM) on the phosphorylation of ERK1/2 relative to control (medium + DMSO 0.1% + phenothiazines 1.0 or 5.0 µM). **(F)** Effect of INR119 (1.0 µM) on the phosphorylation of p38 in comparison to control (medium + DMSO 0.1 % + phenothiazines 1.0 or 5.0 µM). Error bars show standard deviation. Original sample blot is shown in Supplementary Fig. S1 B.
